# Rifampicin-resistance pattern of *Mycobacterium tuberculosis* and associated factors among presumptive tuberculosis patients referred to Debre Markos Referral Hospital, Ethiopia: a cross-sectional study

**DOI:** 10.1186/s13104-016-2328-4

**Published:** 2017-01-03

**Authors:** Wondemagegn Mulu, Bayeh Abera, Mulat Yimer, Tadesse Hailu, Haimanot Ayele, Dereje Abate

**Affiliations:** 1 Department of Medical Microbiology, Immunology and Parasitology, College of Medicine and Health Sciences, Bahir Dar University, Bahir Dar, Ethiopia; 2 Department of Microbiology Laboratory, Debre Markos Referral Hospital, Debre Markos, Ethiopia

**Keywords:** *M. tuberculosis*, Rifampicin, Resistance, Gene Xpert MTB/RIF, Ethiopia

## Abstract

**Background:**

Prevailing data on rifampicin-resistant *M. tuberculosis* is essential for early management of MDR-TB. Therefore, this study was conducted to determine the prevalence of rifampicin-resistant *Mycobacterium tuberculosis* and associated factors among presumptive TB cases in Debre Markos Referral Hospital, Ethiopia.

**Methods:**

A cross-sectional study was conducted from September 2014 to March 2015. Detection of *M. tuberculosis* and resistance to rifampicin was performed using Gene Xpert MTB/RIF assay. Data was collected using structured questionnaire by face to face interview. Logistic regression analysis was computed to determine the associated factors of rifampicin-resistant *M. tuberculosis*.

**Results:**

A total of 505 presumptive TB patients included in the study. The prevalence of *M. tuberculosis* confirmed cases was 117 (23.2%) (95% CI 19.7–27%). It was higher among males (27.9%) than females (17.9%) (AOR: 2.17; CI 1.35–3.49). Of the 117 *M. tuberculosis* confirmed cases, 12 (10.3%) (95% CI 6.0–17.1%) were resistant to rifampicin. Rifampicin-resistant *M. tuberculosis* was noticed in 7 previously treated TB patients (17.1%) and 5 treatment naive patients (6.7%) (AOR: 4.16; CI 1.04–16.63). The prevalence of rifampicin-resistant *M. tuberculosis* was 6 (9.8%) and 6 (11.3%) in pulmonary and extra-pulmonary infections, respectively. Of the 30, MTB/HIV co-infection, 3 (10%) were rifampicin-resistant *M. tuberculosis*.

**Conclusion:**

Rifampicin-resistant *M. tuberculosis* is prevalent in both pulmonary and extra-pulmonary tuberculosis patients. Previous treatment with anti-TB drugs was significantly associated with rifampicin resistance. Therefore, the use of Gene Xpert should be scaled up across the country for rapid detection and management of drug resistant *M. tuberculosis*.

## Background

*Mycobacterium tuberculosis *(*M. tuberculosis*) remains one of the most significant causes of death from an infectious agent [1]. Tuberculosis (TB) remains a major public health problem, accounting more than 9.4 million incident cases and 1.7 million deaths every year, worldwide [2]. World Health Organization (WHO) estimates that 4.5 million people are co-infected with Human Immunodeficiency Virus (HIV) and TB globally [1, 2].

Ethiopia is one of the 22 high burden countries for TB. TB remains one of the leading causes of mortality [3]. According to the 2014 WHO report, the prevalence and incidence of all forms of TB are 211 and 224/100,000 populations, respectively [4]. TB mortality was estimated to be 32/100,000 populations in 2013. Among estimated all new TB cases, 13% are HIV co-infected [3, 4].

The emergence of drug resistance to *M. tuberculosis* has become a significant obstacle for TB control [5]. The emergence and spreading of multidrug (MDR) and extensively (XDR) drug-resistant *M. tuberculosis* complex (MTBC) strains poses significant challenges to TB control [2]. Ethiopia is one of the highest MDR-TB burden countries [3]. In Ethiopia, 2.3% of new TB cases and 17.8% of previously treated TB cases were estimated to have MDR [3]. Studies in Ethiopia reported 4.7–18.3% prevalence of rifampicin-resistant *M. tuberculosis* [6–10]. Mutations in a ‘hotspot’ region of 81 base pairs (bp) of rpoB gene have been found in about 96% of rifampicin (RMP) resistant *M. tuberculosis* [6].

Despite low sensitivity in detection of *M. tuberculosis*, acid-fast staining remains the main diagnostic method in resource-limited settings [11, 12]. Mycobacterial culture is the gold standard and the most sensitive method for TB diagnosis; however, its use in clinical practice is limited due to a slow turnaround time, biosafety requirements, and high cost [11, 12]. In 2011, WHO introduced the wide use of Xpert MTB/RIF assay. It is a fully automated diagnostic molecular test using real-time polymerase chain reaction (PCR) technology to simultaneously detect *M. tuberculosis* and rifampicin resistance mutations in the rpoB gene [13]. The Xpert assay is highly rapid, sensitive and specific in diagnosis of both pulmonary and extra pulmonary TB [8–14]. Furthermore; it was shown to be cost-effective for TB diagnosis compared to microscopy in low and middle income settings [14].

In countries with high burden of TB, rapid detection, continuous surveillance and regular monitoring of drug resistance TB is essential for disease management and earlier treatment initiation. However, there is limited capacity to perform Xpert assay, even from patients suspected of harboring drug-resistant strains with TB/HIV co-infection in Ethiopia. Moreover, documented data on the prevalence of rifampicin resistant *M. tuberculosis* using the newly endorsed method Gene Xpert in our country is limited. Therefore, the aim of this study was to determine the prevalence and associated factors of rifampicin-resistant *M. tuberculosis* among patient’s presumptive for either TB or drug resistant TB (DR TB) in Debre Markos Referral Hospital.

## Methods

### Study design, area and period

A cross-sectional study was conducted from September 2014 to March 2015 at Debre Markos referral Hospital (DMRH). DMRH has more than 147 beds offering different specialized services. It receives patients from the catchment area and referred from different areas of East Gojjam zone. The hospital has TB/HIV clinic as well as MDR-TB ward used for diagnosis and treatment of MDR-TB patients. The Gene Xpert MTB/RIF assay was conducted at DMRH tuberculosis laboratory.

### Sample size

The sample size was determined using single population proportion formula considering 50% expected proportion of rifampicin-resistant *M. tuberculosis* using Gene Xpert MTB/RIF assay, 95% confidence level and marginal error of 5%. Assuming 10% non-response rate, the sample size was: n = 384 + 10% = 384 + 38 = 422. However, 505 patients provided clinical specimen adequately. Any patients attending in the TB clinic of DMRH presumptive for either TB or DR TB were the study population and they were enrolled consecutively.

### Inclusion criteria

Patients presumptive for pulmonary or extra-pulmonary tuberculosis attending in the TB clinic of DMRH and volunteered to participate in the study were included.

### Exclusion criteria

Presumptive patients of pulmonary or extra-pulmonary tuberculosis who provided inadequate specimen for the laboratory analysis were excluded from the study.

### Variables

Rifampicin-resistant *M. tuberculosis* was the dependent variable where as demographic factors, HIV infection status, tuberculosis and treatment related conditions were the independent variables.

### Laboratory procedures

Each eligible patient who signed written consent provided clinical specimens. From each patients presumptive of pulmonary TB, 4 ml of sputum sample was collected. In the case of presumptive extra-pulmonary TB, four ml of either pus, CSF, lymph node aspirate or peritoneal and pleural fluid samples were collected. Samples were immediately processed for Gene Xpert MTB/RIF assay. Clinical samples were diluted and decontaminated and Xpert MTB/RIF assay (Cepheid) was performed according to manufacturer’s instruction. The Xpert® MTB/RIF purifies and concentrates *M. tuberculosis* bacilli from clinical samples. Genomic material isolated from the captured bacteria by sonication and subsequently amplifies the genomic DNA by polymerase chain reaction (PCR). Furthermore, the process identifies all the clinically relevant rifampicin resistance inducing mutations in the RNA polymerase beta (rpoB) gene in the *M. tuberculosis* genome in a real time format using fluorescent probes called molecular beacons.

### HIV testing

Testing for HIV was done according to the current national algorithm recommended by the Federal Ministry of Health of Ethiopia. Two rapid HIV tests, HIV (1 + 2) rapid test strip (KHB) and Stat-Pak were run sequentially. Samples were tested first with KHB. Positive samples were confirmed with Stat-Pak. Discordant results were resolved using a third confirmatory testing kit, HIV-1/2 Unigold Recombinant assay. Pre and post-test HIV counseling was provided for all consenting individuals.

Using a structured questionnaire data was collected by both face to face patient interviews and patients’ clinical record review. The main variables included in the study were age, sex, residence, reason for diagnosis, treatment history, and category of presumptive DR TB and site of tuberculosis.

### Data analysis

Data were analyzed using Statistical Package for Social Sciences (SPSS® 20, USA). Descriptive statistics were used to describe the study participants in relation to relevant variables. Chi-square and logistic regression analysis were computed to identify the associated factors of *M. tuberculosis* and rifampicin-resistance.

Most of the variables were fitted to Chi-square test. Then all variables having a P value of ≤2 in the Chi-square test were further entered into logistic regression model. In the multivariate analysis, backward step wise logistic regression techniques were fitted and confounding were controlled. Variables having P value <0.05 in the multivariate analysis were taken as statistically significant. Adjusted odds ratios with their 95% confidence intervals were calculated. The Hosmer and Lemshow gardens-of-fit test was used to assess whether the necessary assumptions for the application of multiple regressions were fulfilled and P value >0.05 was considered as good fit.

### Quality assurance

Both SPC and PCC internal controls used during Gene Xpert MTB/RIF assay. The specimen was excluded from the analysis if it was an invalid sample for Xpert assay or sample error according to Cepheid package insert. All procedures were done using standard operating methods.

## Results

### Patient characteristics

A total of 505 presumptive TB or DRTB patients participated in the study. Of whom, 188 (37.2%) were presumptive DR TB. Most 265 (52.4%) were males. The age range of participants was 6/12 month to 92 years with mean age of 35.5 year. Majority (55.7%) of participants were urban dwellers. Of the total, 323 (64%) were presumptive for pulmonary TB while 182 (36%) were presumptive for extra-pulmonary TB. Four presumptive DRTB categories were involved in this study: 101 (52.1%) relapse, 62 (32%) new, 26 (13.4%) treatment failure and 4 (2.1%) MDR contact. Prevalence of HIV was 183 (36%) among study participants (Table 1).

**Table 1:** Prevalence of *M. tuberculosis* among presumptive TB patients referred to DMRH using Gene Xpert MTB/RIF assay, 2015

Characters	*M. tuberculosis*		Total N (%)	P value
	Detected N (%)	Not detected N (%)		
Age, years				
≤10	9 (23.7)	29 (76.3)	38 (7.5)	0.017
11–17	7 (29.2)	17 (70.8)	24 (4.8)	
18–30	46 (34.3)	88 (65.7)	134 (26.7)	
31–40	25 (18.7)	109 (81.3)	134 (26.5)	
41–50	15 (15.1)	82 (84.5)	97 (19.2)	
51–60	9 (18)	41 (82)	50 (9.9)	
61–95	6 (21.4)	22 (79.6)	28 (5.5)	
Sex				
Male	74 (27.9)	191 (72.1)	265 (52.4)	0.008
Female	43 (17.9)	197 (82.1)	240 (47.6)	
Residence				
Urban	57 (20.2)	225 (79.8)	282 (55.7)	0.007
Rural	60 (26.9)	163 (73.1)	223 (44.3)	
HIV infection				
Positive	30 (16.6)	153 (83.4)	183 (36)	0.008
Negative	87 (26.9)	235 (73.1)	322 (64)	
Reason for diagnosis				
Presumptive TB	48 (15.1)	269 (84.8)	317 (62.8)	<0.001
Presumptive DR TB	69 (36.7)	119 (63.3)	188 (37.2)	
Treatment history with anti-TB drugs				
Previously treated	41 (31.6)	91 (68.9)	132 (26.2)	0.013
Previously untreated	76 (20.4)	297 (79.6)	372 (73.8)	
Presumptive DRTB				
New	28 (45.2)	34 (54.8)	62 (32)	0.024
Relapse	26 (25.7)	75 (74.3)	101 (52.1)	
Failure	14 (92.3)	12 (7.7)	26 (13.4)	
Lost to follow-up	0	1	1 (0.5)	
MDR-contact	1 (25)	3 (75)	4 (2.1)	
Site of presumptive TB				
Pulmonary	64 (19.8)	259 (80.2)	323 (64)	0.013
Extra-pulmonary	53 (29.1)	129 (70.9)	182 (36)	
Type of specimen				
Respiratory (sputum)	64 (19.8)	259 (80.2)	323 (64)	0.007
Non-respiratory	53 (29.1)	129 (70.9)	182 (36)	
Type of non-respiratory specimen				
Pus	46 (35.1)	85 (64.9)	131 (26)	
Peritoneal fluid	2 (11.8)	15 (88.2)	17 (3.4)	
Lymph node aspirate	1 (7.1)	13 (92.9)	14 (2.8)	
Pleural fluid	3 (23.1)	10 (76.9)	13 (2.6)	
Other	1 (14.3)	6 (83.7)	7 (1.2)	
Total	117 (23.2)	388 (76.8%)	505 (100)	

*MTB M. tuberculosis*, *DR TB* drug resistant tuberculosis

### Prevalence of tuberculosis

The prevalence of *M. tuberculosis* confirmed TB was 117 (23.2%) (95% CI 19.7–27%). The proportion of *M. tuberculosis* was 74 (27.9%) in males and 43 (17.9%) in females. The proportion of *M. tuberculosis* was 48 (15.1%) and 69 (36.7%) among patients presumptive of TB and DR TB, respectively. From 188 presumptive DRTB cases, *M. tuberculosis* was noticed in 28 new (45.2%), 26 relapse (25.7%) and 14 treatment failure (53.8%) cases. *M. tuberculosis* was detected in 64 pulmonary (19.8%) and 53 extra-pulmonary TB cases (29.1%). The rate of MTB/HIV co-infection was 30 (16.6%) (Table 1).

### Rifampicin- resistant *M. tuberculosis*

Of the 117 *M. tuberculosis* cases, 12 (10.3%) were resistant to rifampicin. The proportion of rifampicin-resistant *M. tuberculosis* was 7 (17.1%) among previously treated TB patients and 5 (6.7%) among treatment naïve patients. Of the 69 presumptive DR TB patients, rifampicin resistant *M. tuberculosis* was detected in 3 new (10.7%), 5 relapse (19.2%) and 2 treatment failure (14.3%) cases. Five rifampicin-resistant *M. tuberculosis* was noticed from all patients with MTB/HIV co-infection (17.9%). Rifampicin resistance was noticed in 6 pulmonary (9.5%) and 6 extra-pulmonary tuberculosis cases (11.3%) (Table 2).

**Table 2:** Prevalence of rifampicin-resistant *M. tuberculosis in* each variable among the total *M. tuberculosis* cases using Gene Xpert MTB/RIF assay, DMRH, 2015

Variables	Resistance pattern		P value
	Resistance N (%)	Sensitive N (%)	
Age, years			
≤10	2 (22.2)	7 (77.8)	0.02
11–17	1(14.3)	6 (83.6)	
18–30	3 (6.7)	43 (93.3)	
31–40	0	25 (100)	
41–50	5 (33.3)	10 (66.7)	
51–60	0	9 (100)	
61–92	1 (16.7)	5 (83.3)	
Sex			
Male	8 (11.3)	64 (88.7)	0.77
Female	4 (8.9)	41 (91.1)	
Residence			
Urban	5 (17.9)	53 (91.2)	0.76
Rural	7 (11.9)	52 (88.1)	
HIV infection			
Positive	5 (17.9)	24 (82.1)	0.17
Negative	7 (8)	81 (92)	
Reason for diagnosis			
Presumptive TB	3 (5.9)	49 (94.1)	0.22
Presumptive DR TB	9 (13.8)	56 (86.2)	
Treatment history with anti-TB drugs			
Previously treated	7 (17.1)	34 (82.9)	0.11
Previously untreated	5 (6.7)	71 (93.3)	
Presumptive DR TB			
New	3 (10.7)	25 (89.3)	0.87
Relapse	5 (19.2)	21 (80.8)	
Failure	2 (14.3)	12 (91.7)	
MDR-contact	0	1	
Site of presumptive TB			
Pulmonary	6 (9.4)	58 (90.6)	0.77
Extra pulmonary	6 (11.3)	47 (88.7)	
Specimen type			
Respiratory (sputum)	6 (9.5)	58 (90.6)	0.67
Non-respiratory	6 (11.3)	47 (88.7)	
Type of non-respiratory specimen			
Pus	4 (8.5)	43 (91.5)	
Peritoneal fluid	0	2	
Lymph node aspirate	0	1	
Pleural fluid	2	1	
Total	12 (10.3)	105 (89.7)	

*RIF* rifampicin resistant, *MTB M. tuberculosis*, *DR TB* drug resistant tuberculosis

### Associated factors

Multivariate analysis showed that *M. tuberculosis* infection was significantly associated with male (AOR = 2.17; CI 1.35–3.49), younger age (AOR = 3.2, CI 1.23–8.21), previous TB therapy (AOR = 2, CI 1.03–3.96) and site of TB infection (AOR = 2.19, CI 1.36–3.51). On the other hand, rifampicin-resistant *M. tuberculosis* was significantly associated with previous TB therapy (AOR = 4.16, CI 1.04–16.6). Male patients were 2.17 times more likely to have *M. tuberculosis* infection. TB patients who had previous history of TB therapy were 2 times more likely to have *M. tuberculosis* infection than treatment naïve patients. Moreover, TB patients who were previously treated by anti-TB drugs were 4.2 times more likely to develop rifampicin-resistant *M. tuberculosis* compared to treatment naïve patients (Tables 3, 4).

**Table 3:** Multivariate analysis showing the associated predictors of *M. tuberculosis* in DMRH, 2015

Variables	Gene expert result			
	*M. tuberculosis*		AOR (95% CI)	P value
	Detected	Not detected		
Residence				
Urban	57	225	1.35 (0.86–2.13)	0.19
Rural	60	163		^a^
Sex				
Male	74	191	2.17 (1.35–3.49)	0.001
Female	43	197		^a^
Age, years				
≤10	9	29	3.2 (1.23–8.21)	0.02
11–17	7	17	3.3 (1.08–10.08)	0.036
18–30	46	88	4.6 (1.63–12.71)	0.004
31–40	25	109	2.76 (0.77–9.91)	0.12
41–50	15	82	1.1 (0.46–2.70)	0.82
51–60	9	41	1.2 (0.36–4.02)	0.77
61–92	6	22		^a^
HIV infection				
Positive	30	153	1.36 (0.80–2.3)	0.25
Negative	87	235	^a^	^a^
Reason for examination				
Presumptive TB	48	269	^a^	^a^
Presumptive DR TB	69	119	6.83 (3.55–13.15)	<0.001
Treatment history with anti-TB drugs				
Previously treated	41	91	2.02 (1.03–3.96)	0.04
Previously untreated	76	297		^a^
Site of TB infection				
Pulmonary	64	259	2.19 (1.36–3.51)	0.001
Extra-pulmonary	53	129		

*MTB M. tuberculosis*, *RIF* rifampicin, *AOR* adjusted odds ratio

^a^Reference category, Hosmer–Lemeshow test = 0.92, Pearson Chi-square = 2.55, classification table = 77.4

**Table 4:** Multivariate analysis showing the associated predictors of rifampicin resistant *M. tuberculosis* in DMRH, 2015

Variables	Resistance pattern		AOR (95% CI)	P value
	Resistance	Sensitive		
Age, years				
0–10	2	7	2.3 (0.15–37.67)	0.55
11–17	1	6	1.84 (0.08–44.6)	0.71
18–30	3	43	0.55 (0.04–6.80)	0.64
31–40	0	25		
41–50	5	10	4.3 (0.34–54.99)	0.23
51–60	0	9		
61–92	1	5		^a^
HIV infection				
Positive	5	24	3.2 (0.69–14.96)	0.14
Negative	7	81		^a^
Reason for examination				
Presumptive TB	3	49		^a^
Presumptive DR TB	9	56	0.41 (10.04–4.21)	0.45
Treatment history with anti-TB drugs				
Previously treated	7	34	4.16 (1.04–16.63)	0.04
Previously untreated	5	71		^a^

*DR TB* drug resistant tuberculosis, *AOR* adjusted odds ratio, *CI* confidence interval

^a^Reference category, Hosmer–Lemeshow test = 0.99, Pearson Chi-square = 0.91, classification table = 89.7

## Discussion

In the present study, the prevalence of *M. tuberculosis* infection was similar with reports of South Africa (26%) [15], Northern Nigeria (23%) [16] and India (27.6%) [17]. However, it was lower compared to reports in Nigeria (31.4%) [18] and Pakistan (37%) [19]. The lower proportion rate of confirmed *M. tuberculosis* in the present study compared to other studies is due to the fact that we included presumptive cases to identify *M. tuberculosis* while other studies included identified cases of *M. tuberculosis* to check gene Xpert technique. In contrast, it is higher than studies conducted in other parts of Ethiopia [20–22] and India [23]. The discrepancy might be due to difference in methods of detection of *M. tuberculosis,* community and geographical area.

In this study, the detection rate of *M. tuberculosis* was significantly higher in males than females. Likewise, reports from WHO [24], Ethiopia [7] and Northeast China [25] supports this finding. The reason for this might be due to social and health seeking behavior difference and higher exposure of males to outer environment, smoking and alcoholism [24]. The highest proportion of Gene Xpert positive *M. tuberculosis* cases were seen in the age group of 18–30 years. This is consistent with previous reports in Ethiopia [20–22, 26]. This might be due to more exposure to the outer environment, high work load and wide range of mobility of young people to acquire the TB bacilli as young people have.

In the present study, the proportion of *M. tuberculosis* was significantly higher in presumptive DRTB compared to presumptive TB patients (P < 0.001). This might be due to treatment failure and acquiring of resistant bacilli from drug resistant TB contacts. Moreover, significantly higher proportion of *M. tuberculosis* was found among patients treated with anti-TB drugs compared to treatment naïve patients in the present study. This finding was comparable to a study conducted in Zimbabwe [27].

Rifampicin-resistant *M. tuberculosis* is a serious health problem in the study population. The prevalence of rifampicin-resistant *M. tuberculosis* in this study was in keeping with previous studies in Nigeria [28], North India [29], Iran [30] and Northeast China [25]. However, it was higher than studies observed in Ethiopia [8, 9, 31, 32], Kenya [33], Nigeria [34], Uganda [35] and South of Iraq [36]. In contrast, the proportion of rifampicin-resistant *M. tuberculosis* was lower than reports in other parts of Ethiopia [7, 36] and Chile [37]. The variation could be due to difference in risk for HIV acquisition, exposure to anti-TB drugs and national TB control program. The relative higher proportion of rifampicin-resistant *M. tuberculosis* in our study could be due to the use of rifampicin to treat other conditions. Moreover, rifampicin has several adverse effects which could result in patient non-adherence and hence may lead to the selection of resistant strains.

In the present study, the proportion of rifampicin-resistant *M. tuberculosis* was significantly higher among previously treated patients compared to treatment naive patients which might be due to failure from previous treatment and contact with drug resistant TB patients [26, 38–43]. However, the level of rifampicin resistance among previously untreated cases (6.7%) in the analysis close to the reported prevalence of rifampicin- resistant MTB (10.4%). This finding is a significant relevance in the current global and regional efforts to accurately and timely diagnose MDR-TB with the scale up of molecular technology like Gene Xpert MTB/RIF, providing quick results of rifampicin resistance as a proxy to MDR-TB.

In this study, high prevalence of rifampicin-resistant *M. tuberculosis* was detected among HIV positive cases which are in accordance with a study done in other part of Ethiopia [26] and Cambodia [44]. However, in the present study, there was a lack of association between HIV infection and development of active tuberculosis as well as rifampicin resistance. This was consistent with the results of earlier studies in Ethiopia [39], Tanzania [42], Calabar Nigeria [35] and Brazil [45].

In the present study, the proportion of extra-pulmonary tuberculosis was significantly higher compared to pulmonary tuberculosis; in addition the proportion of rifampicin-resistant *M. tuberculosis* was higher in non-respiratory specimens compared to sputum. This conforms to a study in Cambodia [44]. This demonstrates that rifampicin-resistant extra-pulmonary *M. tuberculosis* infection is a major health problem in resource-limited settings.

The major strength of this study was detection of *M. tuberculosis* and rifampicin resistance using the newly endorsed method Gene Xpert MTB/RIF assay from sputum and non-respiratory specimens. However, the major limitation of this study was determination of the sample size using single population formula which may overwhelm some of the associated factors. This study could not do the level of resistance to other anti-TB drugs and the finding of Gene Xpert was not compared to acid fast bacilli microscopy. Thus the finding of this study should be interpreted with these limitations.

## Conclusions

Rifampicin-resistant *M. tuberculosis* is prevalent both in pulmonary and extra-pulmonary tuberculosis cases in the study area. Previous treatment with anti-TB drugs was significantly associated with rifampicin resistance. The strong association of rifampicin resistance with previous treatment suggests that improved monitoring of treatment to limit the emergence of drug resistant *M. tuberculosis*. Hence, the use of Gene Xpert should be scaled up across the country for rapid diagnosis, management and expanded surveillance of drug-resistant *M. tuberculosis.*
